# Geographic patterns of antibiotic resistance in the human gut microbiome: insights from metagenome-assembled genomes across four Chinese provinces

**DOI:** 10.3389/fmicb.2025.1652757

**Published:** 2025-09-03

**Authors:** Shili Zhou, Huan Liu, Xue Bai, Diyan Li, Tao Wang, Hang Zhong, Wenxia Gao, Jing Sun

**Affiliations:** ^1^School of Pharmacy, Chengdu University, Chengdu, China; ^2^Department of Emergency, Ruijin Hospital, Shanghai Jiao Tong University School of Medicine, Shanghai, China; ^3^Chongqing Academy of Animal Sciences, Chongqing, China; ^4^National Center of Technology Innovation for Pigs, Chongqing, China

**Keywords:** human gut microbiome, antibiotic resistome, metagenome-assembled genomes, regional differences, mobile genetic elements

## Abstract

Next-generation metagenomic sequencing has substantially advanced our understanding of the human intestinal microbiome. Many commensal microbes in the human gut carry a wide array of antibiotic resistance genes (ARGs), collectively forming the gut-associated resistome. In this study, we investigated the gut resistome using metagenomic sequencing. We collected 119 fecal samples from individuals in four Chinese provinces: Yunnan, Guizhou, Sichuan, and Jiangsu. By constructing metagenome-assembled genomes (MAGs) and comprehensive gene catalogs, we aimed to characterize the microbial community structure and assess the distribution of ARGs and mobile genetic elements (MGEs). Our results revealed significant regional differences in gut microbial composition. While Bacillota_A and Actinomycetota were the dominant phyla across all samples, their relative abundances and species-level profiles varied markedly among provinces. Our analysis of ARGs revealed a wide range of antibiotic resistance genes present in the gut microbiota. These ARGs showed uneven distribution across bacterial taxa and among individuals from different regions. For example, ARGs conferring resistance to commonly used antibiotics, such as multidrug agents, peptides, tetracyclines, glycopeptides, and aminoglycosides, were more prevalent in Jiangsu samples than in Sichuan and Yunnan samples, likely reflecting regional differences in antibiotic usage. In MAGs unique to Jiangsu samples, we identified five types of MGEs encompassing 24 subtypes. Among these, transposases (7 subtypes) and recombinases (10 subtypes) were the most abundant. This study offers critical insights into gut resistome compositions and distributions across different populations. Our findings have important implications for public health, microbiota-host interactions, and the development of targeted strategies to mitigate antibiotic resistance.

## 1 Introduction

The global spread of antibiotic resistance has emerged as a major public health challenge in the 21st century (Zhang et al., 2023). To effectively assess the threat posed by antimicrobial resistance (AMR), it is essential to understand the genetic architecture of resistance and to accurately quantify the abundance of antibiotic resistance genes (ARGs) (Bai et al., 2024). Increases in antibiotic production, overuse, and misuse across clinical, agricultural, and environmental settings have contributed to the widespread dissemination of antibiotic-resistant bacteria (ARB) and their associated ARGs. Under selective pressure from antibiotics, ARGs can transfer between bacteria of the same or different species and genera through horizontal gene transfer (HGT), often mediated by mobile genetic elements (MGEs). This process accelerates both the emergence and persistence of antibiotic resistance (Zhai et al., 2024). The intestinal microbiota, often referred to as the “second genome” of the human body, forms a dynamic symbiotic relationship with the host through diverse mechanisms, including metabolite production, immune modulation, and maintenance of the intestinal barrier (Huang et al., 2023; Sheng et al., 2025; Su et al., 2021a; Su et al., 2021b; Yang et al., 2022). Studies have shown that the resistome within the human gut microbiota is closely linked to the evolution of antibiotic resistance in clinical pathogens. Furthermore, ARGs may spread across hosts via environmental vectors such as water and soil, as well as through the food chain, thereby compounding the global resistance crisis (Zhang et al., 2023).

Recent advances in metagenomics have revealed considerable inter-individual variation in gut microbial composition. These differences are shaped not only by host genetic background and dietary patterns but also by environmental and geographical factors (Kim et al., 2024; Liu et al., 2025; Martín et al., 2014). Metagenomic sequencing enables the identification of numerous previously uncharacterized bacterial species within the intestinal microbiota and allows functional characterization at the genomic level (Glendinning et al., 2020; Lewis et al., 2019; Pasolli et al., 2019; Wang et al., 2024b). Studies have demonstrated that methods based on metagenome-assembled genomes (MAGs) and gene catalogs are effective in analyzing the gut microbiota of both humans and animals (Almeida et al., 2021; Chen et al., 2021). MAGs have become essential tools for exploring unknown microbial biodiversity, assessing enzymatic potential, and profiling ecological community structure (Jiménez-Volkerink et al., 2023; Lynes et al., 2023; Rao et al., 2023). To date, reference gene catalogs for the gut microbiome have been established in humans (Almeida et al., 2021) and chickens (Huang et al., 2018), and thousands of MAGs have been generated from diverse hosts, including humans (Pasolli et al., 2019), ruminants (Stewart et al., 2018), chickens (Glendinning et al., 2020), pigs (Wang et al., 2019), and horses (Li et al., 2023). Zeng J’s research group reported a high degree of overlap in ARGs between human gut microbiota and that of farmed animals, such as chickens and pigs. They also identified key bacterial genera, including *Bacteroides* and *Escherichia*, as major contributors to cross-species ARG transmission (Zeng et al., 2019).

Each microbial community in the human body exhibits a distinct structure, shaped by the specific environment it inhabits. In this study, we conducted a comprehensive investigation of the antibiotic resistome within the human gut microbiota using MAGs and gene catalogs derived from 119 human fecal samples. By integrating these genomic resources, we aimed to elucidate the genetic basis of antibiotic resistance, identify potential reservoirs and vectors of ARGs, and explore the ecological and evolutionary dynamics of the resistome. Notably, MAG reconstruction also enables the study of symbiotic fungi and viruses, expanding the scope of microbial community analysis.

## 2 Materials and methods

### 2.1 Sample collection and DNA extraction

We used a total of 119 fecal samples from healthy individuals residing in four Chinese provinces: Sichuan (*n* = 30), Yunnan (*n* = 30), Guizhou (*n* = 29), and Jiangsu (*n* = 30) (Bai et al., 2024). Each participant provided duplicate samples. Detailed sampling information - including species source, province, city, county/district, village/farm, altitude, and GPS coordinates - is provided in Supplementary Table S1. Following collection, we immediately transported all samples on dry ice and stored them at −80°C to preserve microbial DNA integrity for downstream high-throughput sequencing. Total genomic DNA was extracted from each fecal sample using a commercial QIAamp Fast DNA Stool Mini Kit (Qiagen, Hilden, Germany) following the manufacturer’s instructions. The quality and quantity of extracted DNA were assessed using a NanoDrop spectrophotometer and agarose gel electrophoresis.

### 2.2 Metagenomic assembly

We conducted quality control and sequence assembly using the Majorbio Cloud Platform,^1^ a freely accessible online bioinformatics resource.

### 2.3 Metagenome bioinformatics analysis

We first performed adapter trimming and quality filtering on paired-end Illumina reads using fastp (version 0.23.0) (Chen et al., 2018). We removed reads shorter than 50 base pairs or with a quality score below 20. We further refined the reads using SeqPrep, accessible at https://github.com/jstjohn/SeqPrep, and Sickle, available at https://github.com/najoshi/sickle, version 1.33, which performed additional quality trimming and screening. After quality control, we aligned the clean reads to the human reference genome (GRCh38.p13) using BWA^2^ (Li and Durbin, 2009) and removed all host-derived sequences. We then assembled the filtered reads into contigs using MEGAHIT (version 1.1.2) (Li et al., 2015), a *de novo* assembler optimized for large and complex metagenomic datasets.

### 2.4 Metagenomic binning and quality control of MAGs

We conducted genome binning for each sample using three independent tools with default parameters: MetaBAT2 (version 2.12.1) (Kang et al., 2015), MaxBin2 (version 2.2.5) (Alneberg et al., 2014), and CONCOCT (version 0.5.0) (Wu et al., 2016). To generate a high-quality, non-redundant set of MAGs, we integrated the outputs using the standard pipeline of DAS Tool (version 1.1.0) (Sieber et al., 2018). We improved the completeness and reduced the contamination of the resulting MAGs using RefineM (version 0.0.24) (Parks et al., 2017). This tool identifies and removes contigs with aberrant genomic features - such as atypical GC content, divergent tetranucleotide frequency profiles, inconsistent coverage patterns, or conflicting taxonomic assignments. We assessed the completeness and contamination of all MAGs using CheckM (version 1.0.12) (Parks et al., 2015), applying lineage-specific marker genes and default parameters. We retained only MAGs with ≥ 50% completeness and < 10% contamination for subsequent pairwise dereplication. To evaluate genomic similarity, we calculated the average nucleotide identity (ANI) between MAGs using Mash (Ondov et al., 2016) with default settings. We performed dereplication using dRep (version 3.4.2) (Olm et al., 2017), clustering MAGs at a 99% ANI threshold to define strain-level similarity. From each cluster, we selected the highest-quality MAG for downstream analysis. We calculated the coverage of each MAG using CoverM (version 0.6.1) with default parameters (CoverM GitHub repository). Taxonomic classification was performed using GTDB-Tk (version 2.3.0) (Parks et al., 2018) which relies on 120 universal single-copy marker proteins from the Genome Taxonomy Database (GTDB).

### 2.5 Gene prediction and functional annotation

We conducted gene prediction with Prodigal (version 2.6.3) using the -p meta flag to accommodate the metagenomic nature of the data (Hyatt et al., 2010). We annotated the predicted genes against several curated databases, including KEGG (build from August 2023), eggNOG (build from August 2023), CAZy (version 8, build from August 2023), VFDB (build from August 2024), BacMet (version 2.0), CARD (version 3.0.9, build from August 2023), and MGE (MGEs90).

## 3 Results

### 3.1 Samples and metagenomic sequencing data

**Figure 1:**
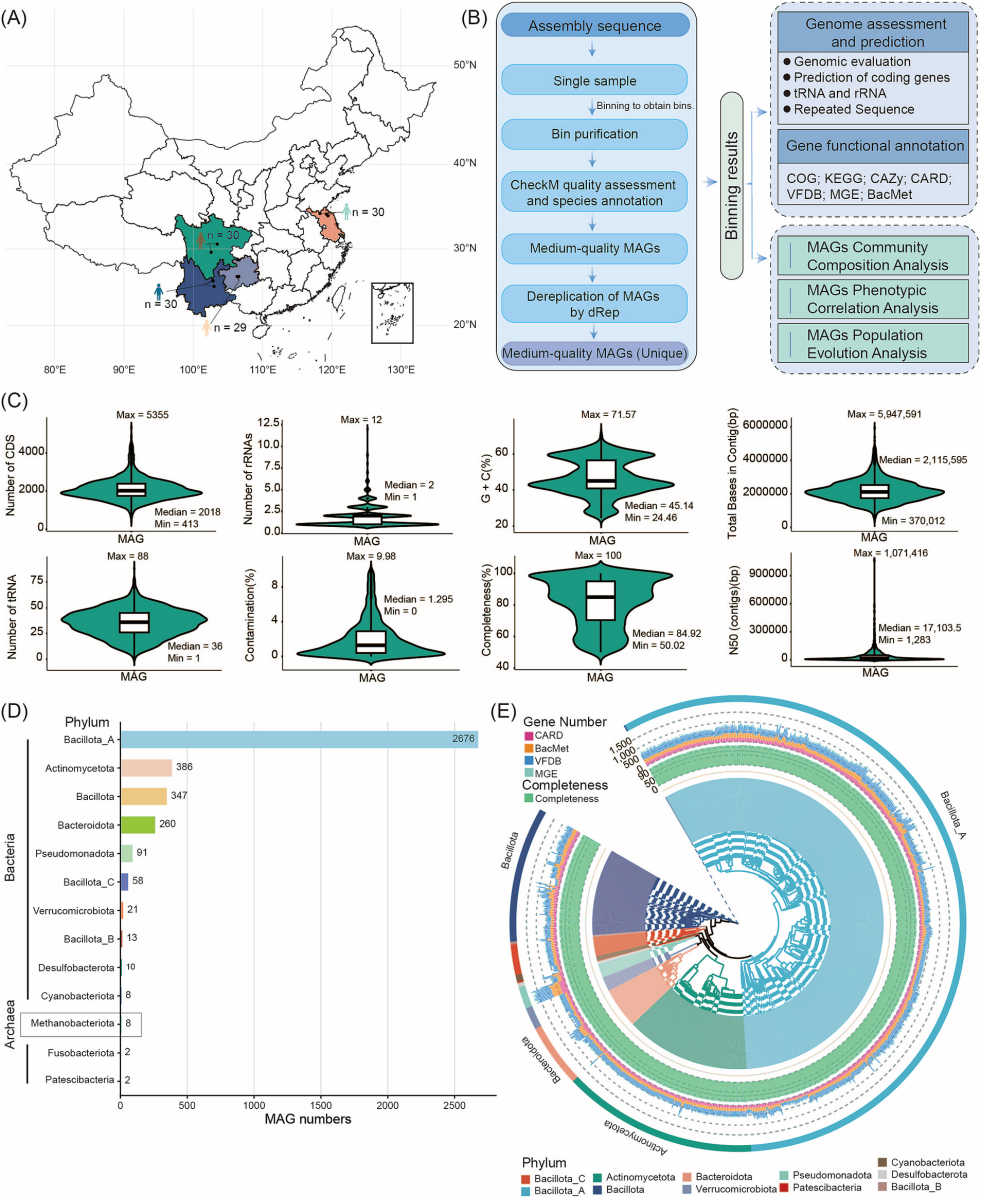
Flowchart of the steps and bioinformatic tools applied in assembling, constructing, annotating, and analyzing reference genomes and microbial gene catalogs. **(A)** Overview of sample collection sites. **(B)** Overview of assembled genomes and functional annotations. **(C)** Assembly statistics for MAGs. Metrics for high- or medium-quality genomes include Genome completeness and contamination, total bases in contig, CDS count, GC%, N50, tRNA number, and rRNAs. **(D)** Number of MAGs detected at the phylum level in descending order (from top to bottom). **(E)** Phylogenetic tree of the high-quality MAG genomes of bacteria. The inner circle of the tree displays the names of the MAGs. Green bars indicate the completeness of the MAGs. The stacked bar chart represents the number of ARGs, MRG/BRG, VFGs, and MGEs genes corresponding to different MAG annotations in the extended CARD/BacMet/VFDB/MGE. The outermost color bands and the background colors of the inner circle of the MAG phylogenetic tree correspond to the different phyla to which the MAGs belong.

To create a resource for studying the human gut microbiome, we performed analysis of metagenome sequencing on fecal samples from 119 individuals across four provinces of China (Supplementary Table S1; Figures 1A,B; Bai et al., 2024). Some samples were from Yunnan Province, where the average altitude is 2,226 meters, the average longitude and latitude are 102.92 and 25.68, respectively, and the annual average temperature is 15.5°C. Some from Jiangsu Province has an average altitude of 21 meters, longitude and latitude of 119 and 34.2, respectively, and an annual average temperature of 14.5°C. Sichuan Province’s average altitude is 603 meters, with average longitude and latitude of 103.14 and 26.85, respectively, and an annual average temperature of 16.17°C. Others from Guizhou Province has an average altitude of 1,409 meters, average longitude and latitude of 105.84 and 26.1, respectively, and an annual average temperature of 14.83°C. Using high-throughput Illumina sequencing, we generated 1.46 Tb of raw data from all 119 samples. After quality control, we retained 1.459 Tb of clean, high-quality data, achieving an effective data quality control rate of 99.33% (Supplementary Table S1).

### 3.2 Assembly of 3,882 MAGs from human gut microbiome sequencing data

We used MetaBAT, CONCOCT, and MaxBin2 for binning the assemblies. After dereplication and quality assessment, the 119 gut fecal metagenomes yielded 3,882 MAGs that met or exceeded medium-quality criteria (completeness ≥ 50% and contamination ≤ 10%) (Supplementary Table S2). Among these MAGs, 2,404 were high-quality genomes (completeness > 80% and contamination < 10%), 942 had > 95% completeness and < 5% contamination, and 24 showed > 97% completeness with 0% contamination (Supplementary Table S2). Detailed metrics, including genome completeness and contamination, total bases in contigs, CDS count, GC content, N50, tRNA count, and rRNAs, are summarized in Supplementary Table S2 and illustrated in Figure 1C. The vast majority of human gut microbes were bacteria (3,874 MAGs), while archaea were rare (eight MAGs). To classify these MAGs, we aligned their sequences against the GTDB. Our analysis assigned the 3,882 MAGs to 13 phyla, 16 classes, 40 orders, 84 families, 310 genera, and 656 species. According to GTDB-Tk assignments, the three dominant bacterial phyla were Bacillota_A (2,676 MAGs, 68.93%), Actinomycetota (386 MAGs, 9.94%), and Bacillota (347 MAGs, 8.94%). Of the eight archaeal MAGs, two belonged to unknown species; all archaeal MAGs were assigned to the Methanobacteriota phylum. The abundance of Bacillota_A and Actinomycetota strains highlights their major role in shaping the human gut microbiota (Supplementary Table S3; Figure 1D). Based on 120 conserved bacterial marker genes and 53 conserved archaeal marker genes from the GTDB database, we constructed a high-quality bacterial MAG phylogenetic tree (with > 95% completeness and < 5% contamination) alongside an archaeal GTDB phylogenetic tree. This provides a robust framework for phylogenetic analysis of the MAGs (Figure 1E, Supplementary Figure S1). By comparing MAGs identified in 119 human samples, we found 272 core MAGs present in at least 90% of samples. These included Blautia_A (123 MAGs), Fusicatenibacter (67 MAGs), and Anaerobutyricum (57 MAGs) (Supplementary Table S4). These core MAGs correspond to genera previously reported in human gut metagenomic data (Kim et al., 2021). Conversely, MAGs with a relative abundance below 1% in 90% of samples were classified as rare (Peng et al., 2021), with only one MAG meeting this criterion.

### 3.3 Analysis of MAG community compositions and correlation analysis of phenotypes

**Figure 2:**
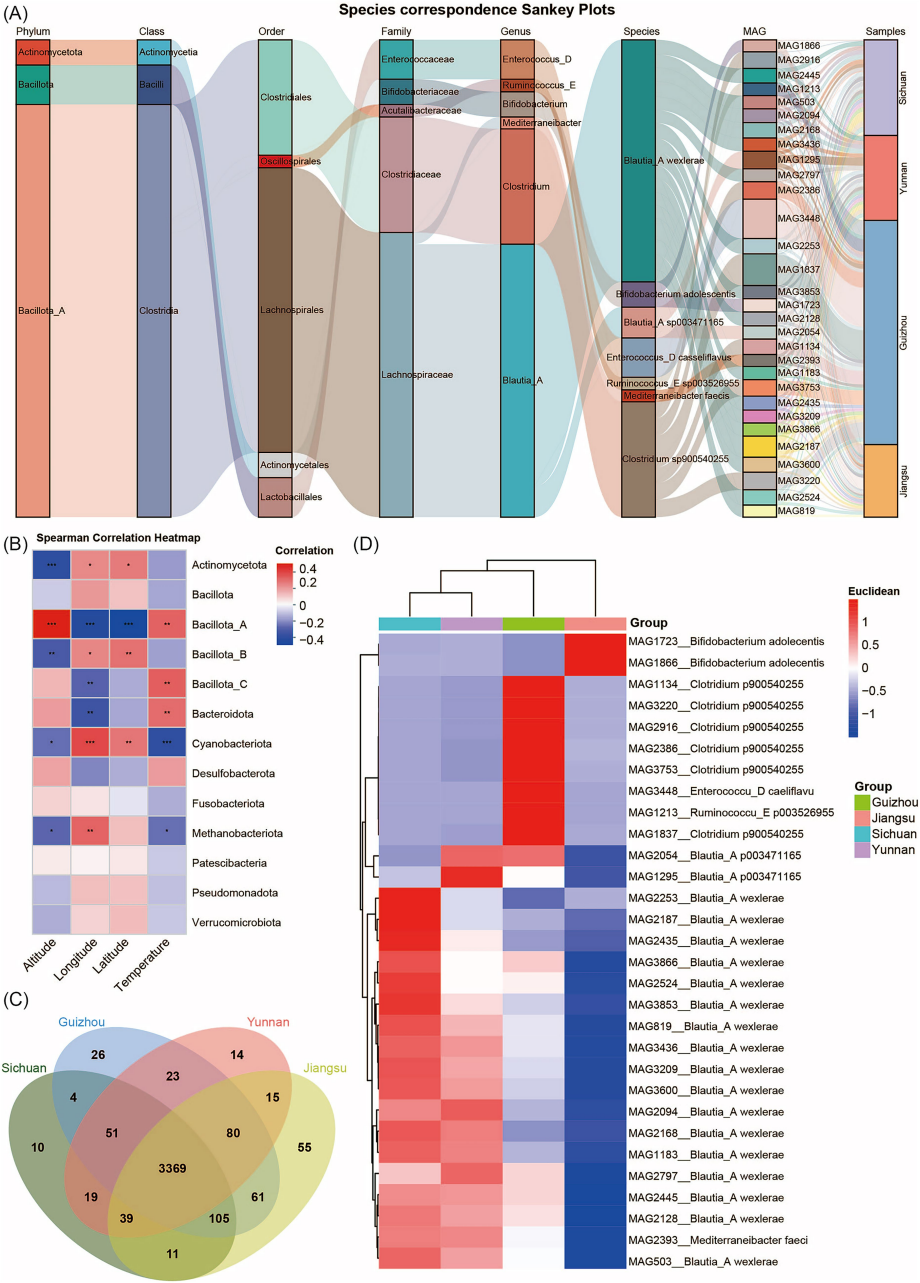
Analysis of MAG community compositions. **(A)** Sankey diagram of community abundance for the top 30 most abundant MAGs. Different columns represent different taxonomic levels. Different color bands within the columns represent species, and the length of the color bands indicate the abundance of the species. Gray connecting lines between the color bands represent the correspondence between species/samples at different levels. **(B)** Spearman correlation heatmap. The x-axis represents different environmental factors, while the y-axis represents the taxonomic units of MAGs at the phylum level. “*” represents *p* < 0.05; “**” represents *p* < 0.01“***” represents *p* < 0.001. **(C)** Venn diagram of MAG distributions across different provinces. **(D)** Heatmap of the top 30 most abundant MAGs clustered among the shared MAGs in Yunnan, Guizhou, Sichuan, and Jiangsu provinces. The pairwise distances among columns were calculated using the Pearson correlation coefficient and subsequently subjected to hierarchical clustering. The right panel clusters these MAGs by province; the blue-to-red gradient indicates increasing MAGs abundance.

We analyzed species abundance across samples using MAG species Sankey diagrams and species heatmaps at multiple taxonomic levels. This analysis clearly illustrated the structural characteristics of community composition and its distribution across different provinces. At the phylum level, Bacillota_A dominated among the top 30 MAGs by abundance. At finer taxonomic resolutions (genus and species), the Sankey diagram revealed more complex branching, with some species showing significant enrichment in specific samples. For example, MAG1213 (*Ruminococcus_E sp003526955*), MAG3448 (*Enterococcus_D casseliflavus*), and MAG1837 (*Clostridium sp900540255*) were significantly enriched in samples from Guizhou Province, likely reflecting the influence of local living environments (Figure 2A; Supplementary Figure S2). Several MAGs showed significant correlations with environmental factors such as altitude, temperature, and geographic coordinates (latitude and longitude). Specifically, the abundance of Bacillota_A MAGs correlated positively with altitude, while Cyanobacteriota MAGs correlated positively with latitude and longitude (Figure 2B). To illustrate the distribution patterns and relative abundance differences of MAGs across provinces, we constructed Venn diagrams and performed principal coordinate analysis (PCoA) for each province’s MAGs, accompanied by heatmaps of the top 30 most abundant common MAGs (Figures 2C,D; Supplementary Figure S3). These analyses revealed that Sichuan harbors 10 unique MAGs, Yunnan has 14 unique MAGs, Guizhou contains 26 unique MAGs, and Jiangsu possesses 55 unique MAGs. Meanwhile, 3,369 MAGs are shared across all four provinces (Supplementary Table S5). Additionally, clustering analysis showed that based on the composition of these 30 highly abundant MAGs, the MAG compositions of the samples from Yunnan and Sichuan show relatively high similarity. Guizhou has a certain degree of similarity with Yunnan and Sichuan, while Jiangsu samples display a distinct community structure. In the Jiangsu samples, the relative abundances of MAG1723 and MAG1866 (*Bifidobacterium adolescentis*) were significantly higher than those in Sichuan, Yunnan, and Guizhou provinces. This phenomenon may be attributed to the fact that the dietary structure in Jiangsu, characterized by light and fresh flavors with frequent consumption of rice, fish, shrimp, poultry, as well as higher intake of soy products and dairy products, provides abundant nutritional substrates such as proteins and lactose for *Bifidobacterium adolescentis.* Additionally, the flat terrain, abundant drinking water sources, and relatively good water quality in Jiangsu create a favorable growth environment for this bacterium. In contrast, the heavier-flavored diets in Sichuan, Yunnan, and Guizhou—rich in spicy foods—and the higher mineral content in the water of these regions may alter the intestinal microenvironment, thereby inhibiting the proliferation of *Bifidobacterium adolescentis*. In Guizhou samples, the relative abundance of MAG3448 (*Enterococcus_D casseliflavus*) and MAG1837 (*Clostridium sp900540255*) was significantly higher than in other provinces, potentially reflecting unique environmental conditions or host dietary habits in this region.

### 3.4 Functional annotation using COG, KEGG, BacMet, VFDB, and CAZy databases

**Figure 3:**
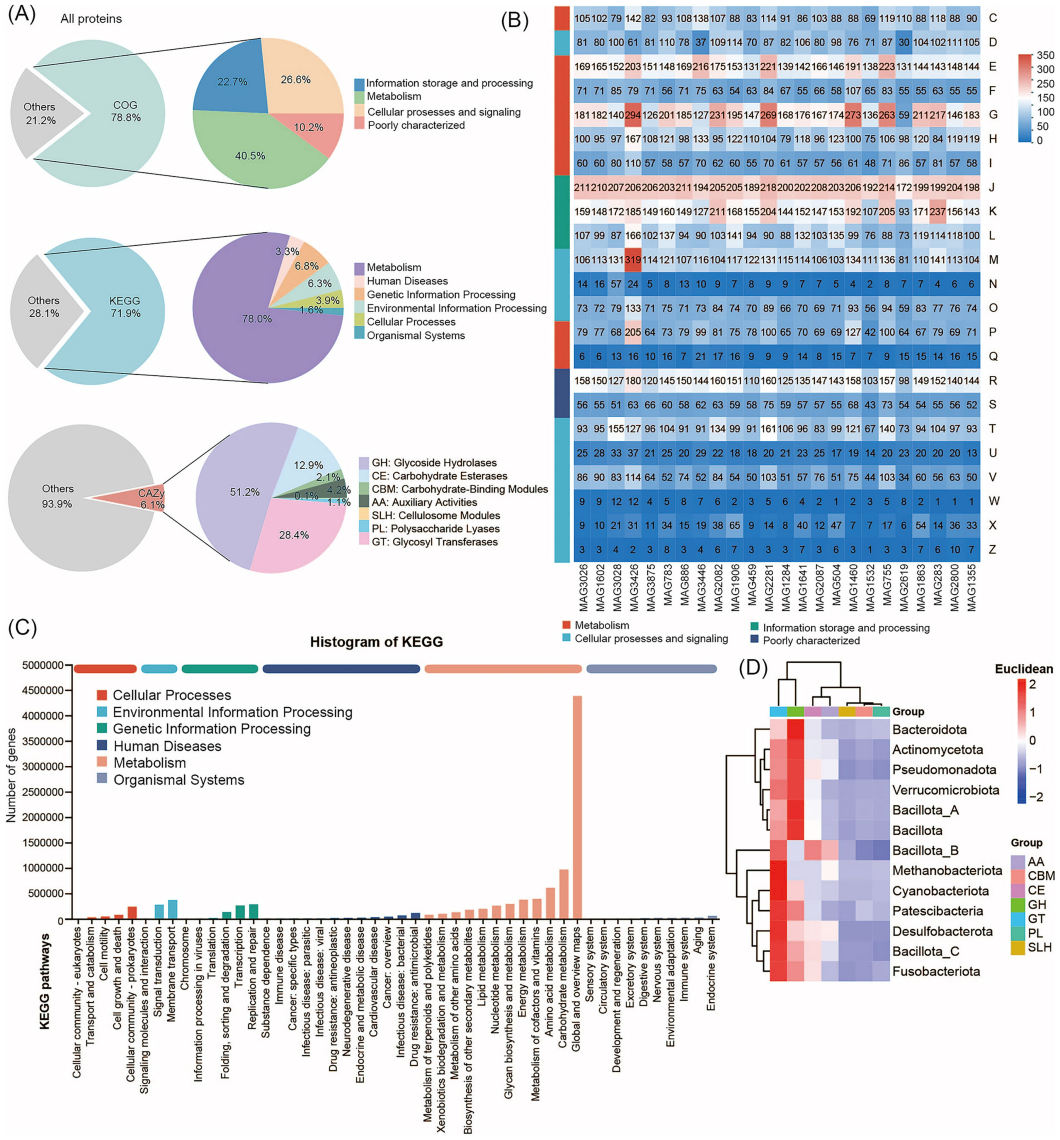
Functional annotation using COG, KEGG, and CAZy databases. **(A)** Functional annotations of human microbial proteins. Annotation results are obtained using COG, KEGG, and CAZy. **(B)** COG classification statistical heatmap. The horizontal axis represents different MAG genomes, and the vertical axis represents the number of genes for different functions. Refer to the legend for the functional descriptions of specific COG types. **(C)** Pathway classification statistical bar chart. The vertical axis represents the level 2 classification of KEGG pathways, and the horizontal axis represents the number of genes annotated with this classification. Colors of different bars represent the level 1 classification of KEGG pathways. The rightmost bar indicates the number of genes under different level 1 classifications. **(D)** Heatmap of the distributions of CAZy. The horizontal axis represents 7 different kinds of CAZy, and the different colors of the vertical axis represent different bacterial taxonomic information.

Next, we constructed an integrated human microbial gene catalog comprising 3,882 nonredundant genes and analyzed the proteomic content and functional potential of the human metagenomes by searching against the COG, KEGG, BacMet, VFDB, and CAZy databases. The 3,882 medium- and high-quality MAGs contained a total of 8,156,198 predicted proteins. Among these, 78.78% (6,425,193 proteins) were predicted to have at least one COG function, 71.87% (5,861,652 proteins) had at least one KEGG function, and 6.77% (299,384 proteins) had at least one CAZy function (Figure 3A; Supplementary Table S6). The COG functional classification revealed that the MAGs were annotated into four broad categories: Cellular Processes and Signaling, Information Storage and Processing, Metabolism, and Poorly Characterized. These categories encompassed 25 specific COG types, with Metabolism representing the largest proportion (Supplementary Table S7). Functional annotation of predicted coding genes from the 24 high-quality MAGs (each with > 97% completeness and 0% contamination) further demonstrated that genes related to Translation, Ribosomal Structure and Biogenesis, and Carbohydrate Transport and Metabolism were predominant, as determined using the COG database (Figure 3B). The KEGG annotation results, shown in Figure 3C, classified protein functions into six metabolic systems. Proteins associated with metabolism accounted for approximately 78.02% of the total. Within this category, genes related to Global and Overview Maps and Amino Acid Metabolism were most abundant (Supplementary Table S8). Together, these findings are consistent with the COG-based results and indicate that the functional landscape of the human gut microbiome is dominated by metabolic processes, with amino acid metabolism emerging as the most prominent metabolic pathway.

We annotated the data using the CAZy database. The results show that the 299,384 CAZy proteins included 153,209 glycosyl hydrolases (GH), 84,958 glycosyl transferases (GT), 6,420 carbohydrate-binding modules (CBM), 38,583 carbohydrate esterases (CE), 3,292 polysaccharide lyases (PL), 12,523 proteins with auxiliary activity (AA), and 399 cellulosome modules (Supplementary Table S9). We found that Glycosyl Transferases were the predominant enzymes across almost all MAGs, followed by Glycoside Hydrolases, Carbohydrate Esterases, and proteins with Auxiliary Activities. These proteins were unevenly distributed across the genomes of the taxa we identified. For example, GH and GT proteins were particularly enriched in Bacillota_A and Bacteroidota (Figure 3D). We further annotated the 24 high-quality MAGs (each with > 97% completeness and 0% contamination) using the BacMet and VFDB databases to analyze resistance genes and virulence factors in the human gut microbiome. In total, we detected 124 resistance genes and 381 virulence factors (Supplementary Table S10). The identified virulence factors primarily included components involved in nutrition and metabolism, immune modulation, adherence, regulation, and other functions (Supplementary Figure S4). The most abundant resistance gene was Cu (metal), with a frequency of 6.8%. Analysis of unique MAGs from Jiangsu Province revealed the highest diversity and abundance of resistance genes. The distribution of resistance genes in the gut microbiome also showed distinct patterns. At the phylum level, Bacillota_A and Bacteroidota were the primary hosts of these genes (Supplementary Figure S5).

### 3.5 Antibiotic resistance gene profiling

**Figure 4:**
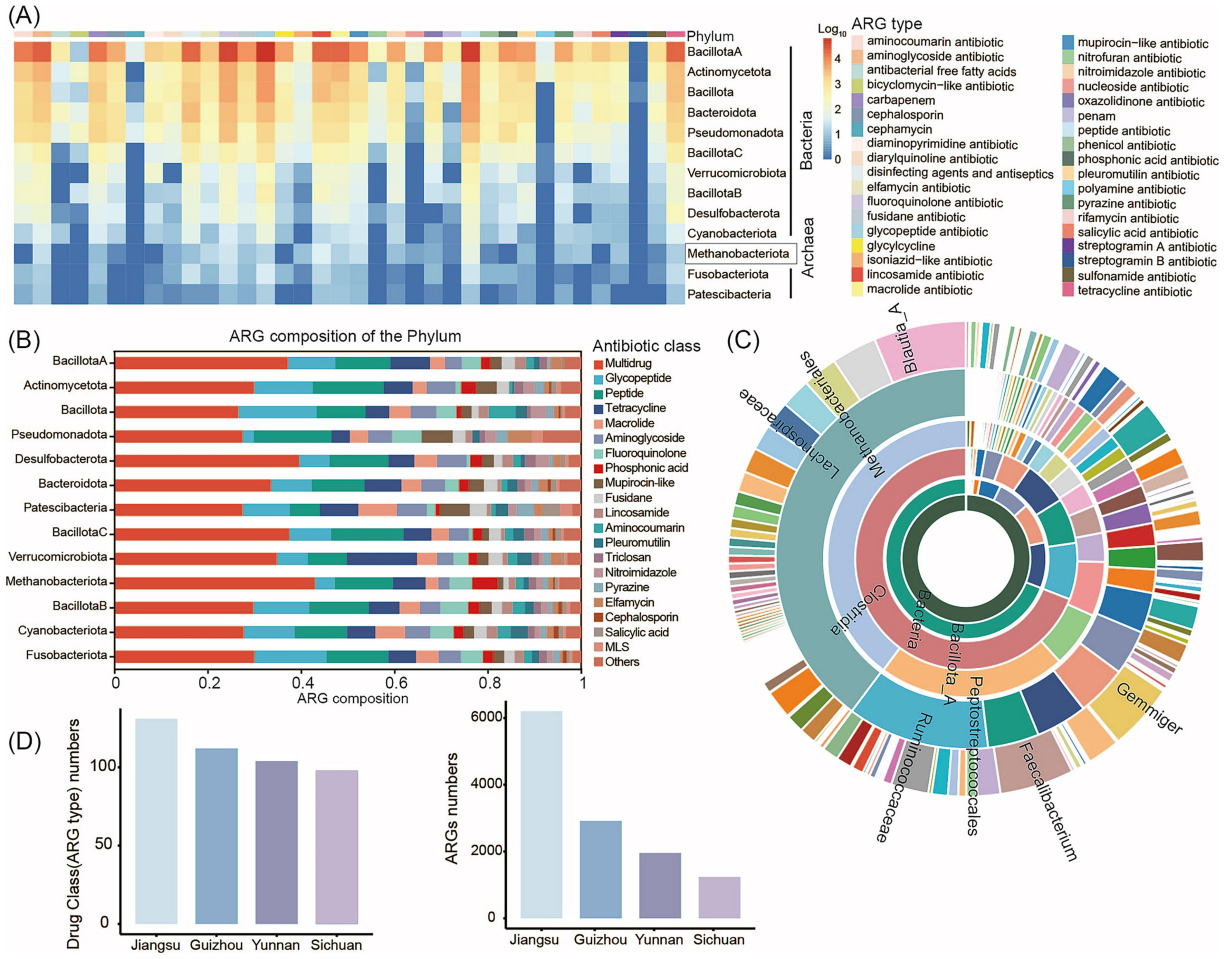
Profiling of the antibiotic resistome in the human gut microbiome. **(A)** Heatmap of the ARG distribution. Color from blue to red represents an increasing number of ARG. Color bands at the top of the heatmap represents the resistance drug corresponding to each column of ARG. **(B)** Antibiotic resistance gene prediction and classification statistics chart. The vertical axis represents different phyla, while the horizontal axis shows the percentage distribution of ARG annotations to gene numbers. Different colored blocks within the bars correspond to different antibiotic classes. **(C)** Number of ARGs in MAGs at different taxonomic levels. The inner to outer portions represent the kingdom level to the genus level. The color represents different species in different taxonomic groups. **(D)** ARG gene count and drug class (ARG type) numbers of MAGs specific to human intestinal microbiota in different regions. The abscissa represents the 4 regions (Sichuan, Yunnan, Guizhou, and Jiangsu).

To characterize human intestinal ARGs, we analyzed the distribution of ARGs across 3,882 MAGs of at least medium-quality standard from 119 human gut samples. We performed ARG profiling using the CARD database. In total, we identified 159 unique ARG types spanning 35 drug resistance classes within the human gut MAGs. Recent studies of the human gut microbiome have reported a link between antibiotic use and the prevalence of ARGs in the microbiome (Kim et al., 2024). Consistent with these findings, we observed that Bacillota_A and Actinomycetota harbored the greatest numbers of ARGs. We also detected ARGs in eight archaeal taxa, including Methanobacteriota (Figure 4A; Supplementary Table S11). Multidrug, glycopeptide, aminoglycoside, and tetracycline resistance genes were prevalent in the human intestine (Figure 4B). Among bacterial genera (considering only those represented by ≥ 5 genomes), *Blautia_A*, *Faecalibacterium*, and *Gemmiger* contained the highest numbers of ARGs per genome (Figure 4C). Multidrug resistance genes were the most abundant ARGs in the human gut, followed by tetracycline resistance genes. The high abundance of tetracycline resistance genes is of particular concern, given that tetracycline has been widely used to treat bacterial infections, including respiratory tract diseases. This widespread presence of tetracycline resistance genes may therefore have negative implications for human health.

Furthermore, all 3,882 MAGs contained five or more ARGs, indicating that ARGs are widespread throughout the human gut microbiome. Notably, we identified an *Escherichia coli* strain (MAG1203) harboring 84 unique ARG types spanning 27 drug resistance classes (Supplementary Figure S6; Supplementary Table S12). This strain carried a broad array of resistance genes targeting multidrug antibiotics, peptide antibiotics, tetracyclines, glycopeptides, phosphonic acids, aminoglycosides, and fluoroquinolones. Given the well-documented pathogenicity of *E. coli*, this finding suggests that this strain may represent a potential drug-resistant superbug. In addition, we detected a substantial number of ARGs in MAG3398 (*Anaerostipes hadrus*), MAG94 (*Faecalibacterium duncaniae*), and MAG147 (*Anaerobutyricum hallii*), species that are widely reported to exhibit probiotic properties (Supplementary Table S12). While the presence of these ARGs could potentially facilitate future isolation and culture of these strains, it also raises concerns regarding the possible adverse impacts of these resistance genes on the host gut microbiome.

We next performed pairwise comparisons of ARG profiles in gut-specific MAGs from populations across four Chinese provinces (Sichuan, Yunnan, Guizhou, and Jiangsu). Our analysis revealed that both the number of ARGs and the number of drug resistance classes were highest in samples from Jiangsu Province, with ARG abundance exceeding that observed in Sichuan, Yunnan, and Guizhou by more than twofold (Figure 4D; Supplementary Table S13). This pattern may reflect the fact that Jiangsu Province is economically developed, with high population mobility and more extensive use of antibiotics in both medical and agricultural settings. These factors likely contribute to the greater abundance and diversity of resistance genes observed. In contrast, although Sichuan and Yunnan differ in genetic backgrounds and environmental conditions, their socioeconomic factors and lifestyles - including dietary habits and hygiene practices - are relatively similar. In addition, there is a moderate degree of population movement and interaction between these regions. Resistance genes can disseminate across regions through interpersonal contact and transmission, which may explain the similar number and diversity of ARGs and drug resistance classes observed in the gut microbiomes of individuals from these provinces. Furthermore, the spread of ARGs can occur between different bacterial species through horizontal gene transfer, which may further exacerbate the growing challenge of antibiotic resistance.

### 3.6 Mobile genetic elements related to antibiotic resistance genes

**Figure 5:**
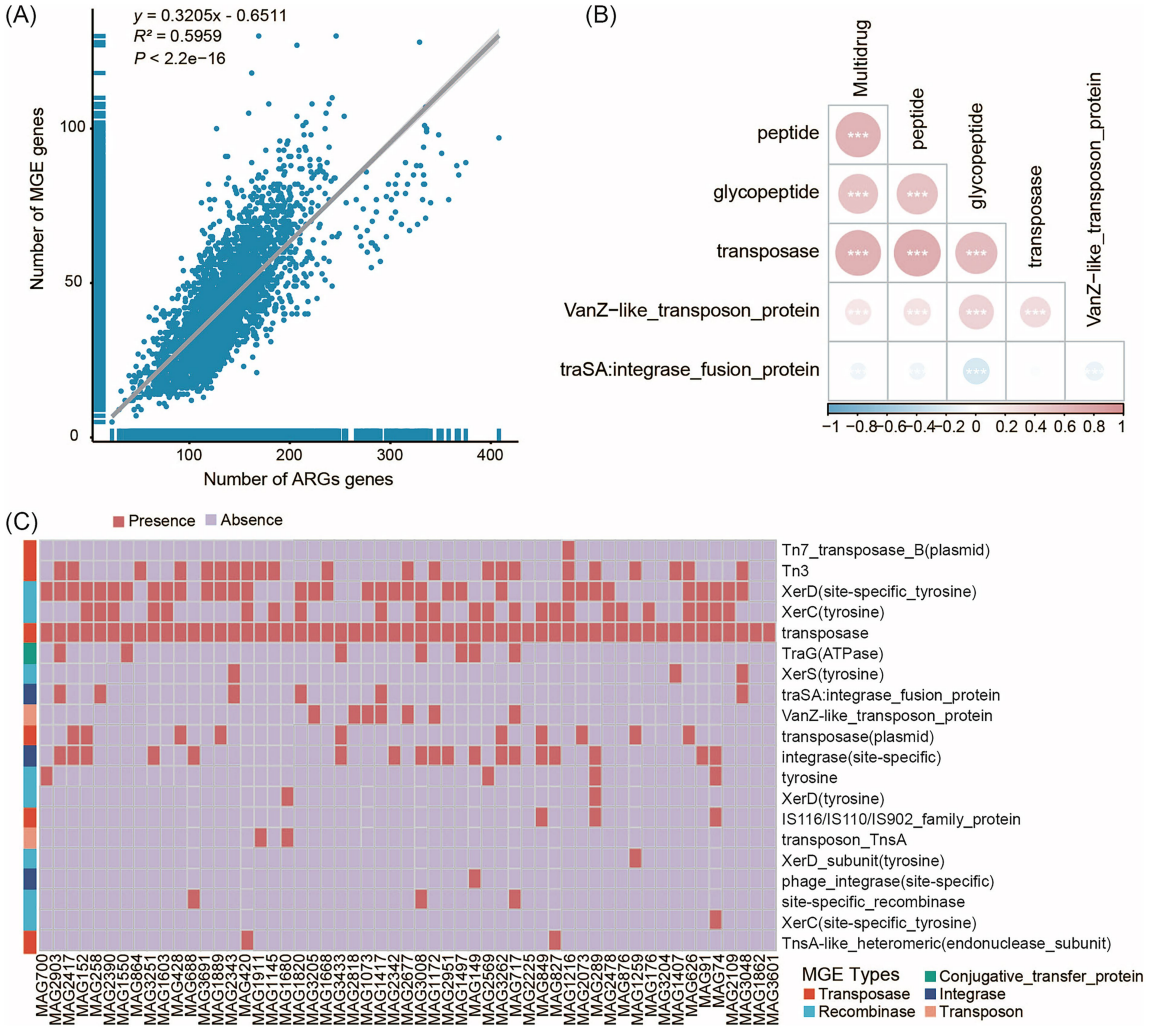
The correlation between ARGs and MGEs. **(A)** The overall correlation between ARGs and MGEs in 119 human fecal samples. **(B)** Relevant heat maps of Antibiotic class and MGEs. **(C)** Classification results of MGEs in unique MAGs of the Jiangsu province. The x-axis represents the genome MAG IDs, while the y-axis shows the MGEs. The red legend “Presence” indicates the annotation of MGEs, and the light purple legend “Absence” indicates no annotation of MGEs. Different colored blocks in the y-axis clustering tree represent different MGE types.

The association between resistance genes and MGEs, such as plasmids and transposons, is a key factor in evaluating the potential for ARG dissemination within microbial communities. HGT, facilitated by MGEs, is the primary mechanism by which bacteria acquire ARGs. We employed Spearman analysis and linear regression analysis to evaluate the correlation between MGEs and ARGs. The results revealed a significant linear correlation between MGEs and ARGs (y = 0.3205x - 0.6511, R^2^ = 0.5959, *p* < 2.2e−16), indicating that the increase in MGEs may facilitate the dissemination of ARGs (Figure 5A). This correlation was further confirmed by a correlation heatmap (Supplementary Figure S7), which showed that among different types of MGEs, transposase exhibited significant positive correlations with Multidrug (Figure 5B). To investigate the potential mechanisms driving ARG dissemination across Sichuan, Yunnan, Guizhou, and Jiangsu provinces, we characterized the number of MGEs present in the unique MAG populations from these four regions (Supplementary Table S13). Our analysis identified 3,559 MGEs (Table S1) in gut microbiota representatives spanning the following taxa: 46 strains of Actinomycetota (7 species), 72 strains of Bacillota (12 species), 183 strains of Bacillota_A (41 species), 3 strains of Bacillota_B (1 species), 13 strains of Bacillota_C (3 species), 45 strains of Bacteroidota (8 species), 25 strains of Cyanobacteriota (4 species), 11 strains of Methanobacteriota (3 species), 3 strains of Patescibacteria (1 species), 22 strains of Pseudomonadota (2 species), and 6 strains of Verrucomicrobiota (2 species).

We identified five types of MGEs (comprising 30 subtypes): conjugative transfer proteins, integrases, recombinases, transposases, and transposons. The majority of MGEs (2,349 of 3,559) originated from Bacillota_A and Bacillota, which are known to play important roles in the gut microbiota of healthy adults (Do et al., 2024). Recombinases (12 subtypes) and transposases (10 subtypes) were the predominant MGE types identified. The number of MGE subtypes varied among samples, with Jiangsu Province exhibiting a significantly higher total number of MGE subtypes compared to Yunnan Province. In the MAGs unique to samples from Jiangsu Province, we identified five types of MGEs spanning 24 subtypes. Transposases (7 subtypes) and recombinases (10 subtypes) were the predominant MGE types (Figure 5C). The major subtypes of integrases, transposases, and recombinases were integrase (site-specific), Tn3, and XerD (site-specific tyrosine), respectively. We also identified both shared and unique MGEs among the unique MAG populations from the four provinces. Specifically, five unique MGE subtypes were detected in Jiangsu Province - Tn7_transposase_B (plasmid), transposase-like_protein_B (plasmid), transposon_TnsA, transposon-related_protein, and XerD_subunit (tyrosine) - while two unique subtypes were found in Guizhou Province - transposon_Tn21_modulator_protein (plasmid) and XerD (site-specific).

Previous studies have shown that the physical proximity of MGEs and ARGs (< 5.0 kb) can strongly promote HGT. To assess the potential for horizontal transfer of ARGs at the strain level, we further analyzed the distribution of MGEs in three *Escherichia coli* MAGs (MAG38, MAG2283, and MAG1203), each of which carried more than 100 ARGs (Supplementary Table S14). These three MAGs contained 6, 8, and 7 MGEs, respectively. Notably, transposase elements - found at multiple locations within each MAG - frequently co-occurred with various ARGs on the same contigs. For example, in MAG1203, we identified a gene cluster located within a 120 kb region of a single contig containing 13 ARGs and 4 MGEs, including 4 transposases (Supplementary Figure S8). This finding suggests a high risk of horizontal ARG transfer facilitated by MGEs in these strains.

## 4 Discussion

The human gut microbiome is now recognized as a complex ecosystem that plays a vital role in human health and disease (Kawano-Sugaya et al., 2024). Recent efforts have generated genome collections of human gut microbes based on MAGs, including the Unified Human Gut Genome (UHGG) (Almeida et al., 2021) and HumGut (Hiseni et al., 2021). However, these collections typically use only a single representative genome per species, which can lead to substantial gene loss. Moreover, even genomes of the same microorganism can vary significantly when isolated from different environments (Zheng et al., 2024). Recent studies have greatly expanded the catalog of known species within the human gut. Three independent efforts have reconstructed between 60,000 and 150,000 MAGs from public human microbiome datasets, with the majority representing species that lack cultured representatives (Almeida et al., 2019; Nayfach et al., 2019; Pasolli et al., 2019). Pasolli et al. (2019) assembled over 153,000 high- and medium-quality draft genomes from metagenomic samples, demonstrating the capability of high-throughput metagenomic genome mining. Their research mainly focused on the extensive diversity of the human microbiome across different ages, geographical regions, and lifestyles. Almeida et al. (2021) presented a unified catalog containing 204,938 reference genomes of the human gut microbiome, aiming to comprehensively document the genomes of the human gut microbiome. Integrating these independent resources into a unified, non-redundant human gut genome dataset is essential for advancing future microbiome research. Both of these studies took the gut microbiomes of populations from multiple global regions as research objects, constructed large-scale genomic collections (such as the UHGG database), but focused more on the large-scale construction of metagenomic resources and the analysis of common characteristics across populations. In contrast, we systematically characterized the antibiotic resistome of gut microbiomes from populations in four Chinese provinces (Sichuan, Yunnan, Guizhou, and Jiangsu) through metagenomic sequencing and MAG-based analysis. We focused on regional-specific differences and, through comparative analysis of populations with significant differences in geographical isolation, dietary cultures, and medical practices, revealed the structural features of the gut microbial communities, mapped the distribution patterns of ARGs, and elucidated resistance transmission mechanisms mediated by MGEs. These findings provide new insights into the regional heterogeneity of human gut resistomes.

We observed significant differences in gut microbial community structures among individuals from different provinces. Previous studies have shown that unifying human gut genomes - including both MAGs and isolate genomes - provides valuable insights into the richness, diversity, and cultivability of the gut microbiome across multiple taxonomic and functional levels (Almeida et al., 2021). Using the assembled genomes to analyze gut microbiome functions, we found that although Bacillota_A and Actinomycetota were the dominant phyla, their relative abundances and species compositions varied markedly across regions. For example, the Jiangsu population exhibited significantly higher relative abundances of MAG1723 and MAG1866 (*Bifidobacterium adolescentis*) compared to populations from southwestern regions (Yunnan and Sichuan). In contrast, MAG3448 (*Enterococcu_D caeliflavu*) and MAG1837 (*Clostridium p900540255*) were specifically enriched in samples from Guizhou. These regional differences likely reflect a combination of factors, including geographic environment, dietary habits, lifestyle, and patterns of antibiotic use. COG functional classification revealed that human gut microbial genomes are broadly distributed across numerous biological functions. These span basic cellular processes - such as transcription, translation, and energy metabolism - as well as functions related to environmental adaptation and host interactions, including secondary metabolite synthesis and ion transport. KEGG functional annotation further revealed the specific roles of genes within metabolic pathways and functional modules, demonstrating that gut microbes form complex metabolic networks and collaborative mechanisms in amino acid metabolism, carbohydrate metabolism, energy metabolism, and global overview maps. These functional annotation results not only offer important insights for in-depth investigations of the metabolic characteristics of human MAGs, but also establish a foundation for understanding the interactions between gut microbes and their hosts, as well as the mechanisms underlying gut health and disease. We also identified a large number of resistance genes and virulence factors, whose types, quantities, and distribution patterns varied among individuals and were closely linked to host and environmental factors. The presence of these resistance genes may pose significant public health risks. Future studies should integrate experimental validation with advanced bioinformatics analyses to further elucidate the actual roles and regulatory mechanisms of these functional genes, thereby providing a theoretical basis for developing gut microbiome-based health intervention strategies.

Our findings confirm the presence of multiple ARGs within the gut microbiota, which are unevenly distributed across bacterial taxa and among individuals. The widespread occurrence of multidrug resistance genes may complicate the treatment of intestinal infections and further threaten public health. This study revealed significant differences in ARG resistance levels to commonly used antibiotics between the Jiangsu and Sichuan/Yunnan regions, clearly reflecting regional patterns of antibiotic use. Our ARG analysis showed that both the number of ARGs and the number of drug classes (ARG types) were highest in Jiangsu Province. The ARG count in Jiangsu was more than double that observed in Sichuan, Yunnan, and Guizhou. This disparity may be linked to the higher annual usage of antibiotics, such as tetracyclines and glycopeptides, in medical prescriptions in Jiangsu compared to the southwestern provinces. Variations in medical standards, the scale of livestock industry development, and public awareness and habits regarding antibiotic use across regions likely contribute to the long-term exposure of gut microbes to different levels of antibiotic selective pressure, thereby shaping distinct microbial community structures and resistomes. These findings underscore the need to carefully consider regional factors when studying the gut microbiome and provide a foundation for developing region-specific public health strategies. MGEs, such as plasmids and bacteriophages, can transfer between bacterial hosts and often serve as vectors for ARGs, thereby facilitating antimicrobial resistance in bacteria (Brito, 2021; McInnes et al., 2020). In this study, we identified various types of MGEs within MAGs unique to Jiangsu province samples, with transposases and recombinases being the most prevalent. These MGEs can mediate the horizontal transfer of ARGs between different bacterial species, accelerating the spread of resistance genes within the gut microbial community. Variations in the diversity and abundance of MGEs may contribute to the uneven distribution of ARGs across regions and individuals. Moreover, the presence of MGEs may drive the rapid evolution and adaptation of microbes under antibiotic pressure, further intensifying the challenge of antibiotic resistance. Therefore, intervention strategies targeting MGEs could represent a promising approach to controlling the spread of antibiotic resistance. Such strategies may include the development of novel drugs or biological agents that inhibit MGE activity or block their transfer pathways.

However, this study has certain limitations. First, the samples were collected from only four provinces in China. Although these regions encompass diverse geographical areas and living environments, they may not fully capture global microbial diversity. Future studies should broaden sampling efforts to include additional regions and populations to achieve a more comprehensive understanding of the gut microbiome antibiotic resistome. Second, we primarily focused on the functional annotation of MAGs unique to each province. Further research should investigate the specific functions of ARGs and MGEs within provincial MAGs and their effects on host health. Additionally, this study did not extensively examine the complex interactions between host factors - such as age, gender, and underlying health conditions - and the gut microbiome antibiotic resistome. In future research, we can combine multi-omics techniques such as single-cell transcriptomics (Leng et al., 2024; Wang et al., 2024a; Wang et al., 2025) to explore the relationship between the metagenome, its resistance genes, and the host phenotype.

To sum up, the extensive application of MAGs from human fecal samples can greatly enhance our understanding of the human gut microbiome. From a public health perspective, comprehensive analyses of the microbiome, mobilome, and resistome offer valuable strategies to address the escalating challenge of antimicrobial resistance (Baquero et al., 2019; Djordjevic et al., 2024). Given the observed spread of antimicrobial resistance across different provinces, it is essential to understand the reservoirs and transmission pathways of ARGs (Fredriksen et al., 2023; Kawano-Sugaya et al., 2024). Our study provides an exhaustive reference genomic dataset for the human gut microbiota and underscores the complex interactions between the host and the gut microbiome. Future research should address current limitations, further investigate the mechanisms underlying the formation and regulation of antibiotic resistance within the intestinal microbiome, and develop more effective strategies to protect public health and combat antibiotic resistance.

## Data Availability

The data presented in the study are deposited in the NCBI BioProject repository, accession number PRJNA1301879.

## Footnotes

^1^ www.majorbio.com

^2^http://bio-bwa.sourceforge.net/, version 0.7.9a
