# Supplementary material for: Geographic patterns of antibiotic resistance in the human gut microbiome: insights from metagenome-assembled genomes across four Chinese provinces
Source: Front Microbiol. 2025 Sep 3;16:1652757. doi: 10.3389/fmicb.2025.1652757 (PMC12442556; doi:10.3389/fmicb.2025.1652757)
Supplement: Supplementary file 1 [file Supplementary_file_1.docx]

Supplementary Material

# Supplementary Figures

**
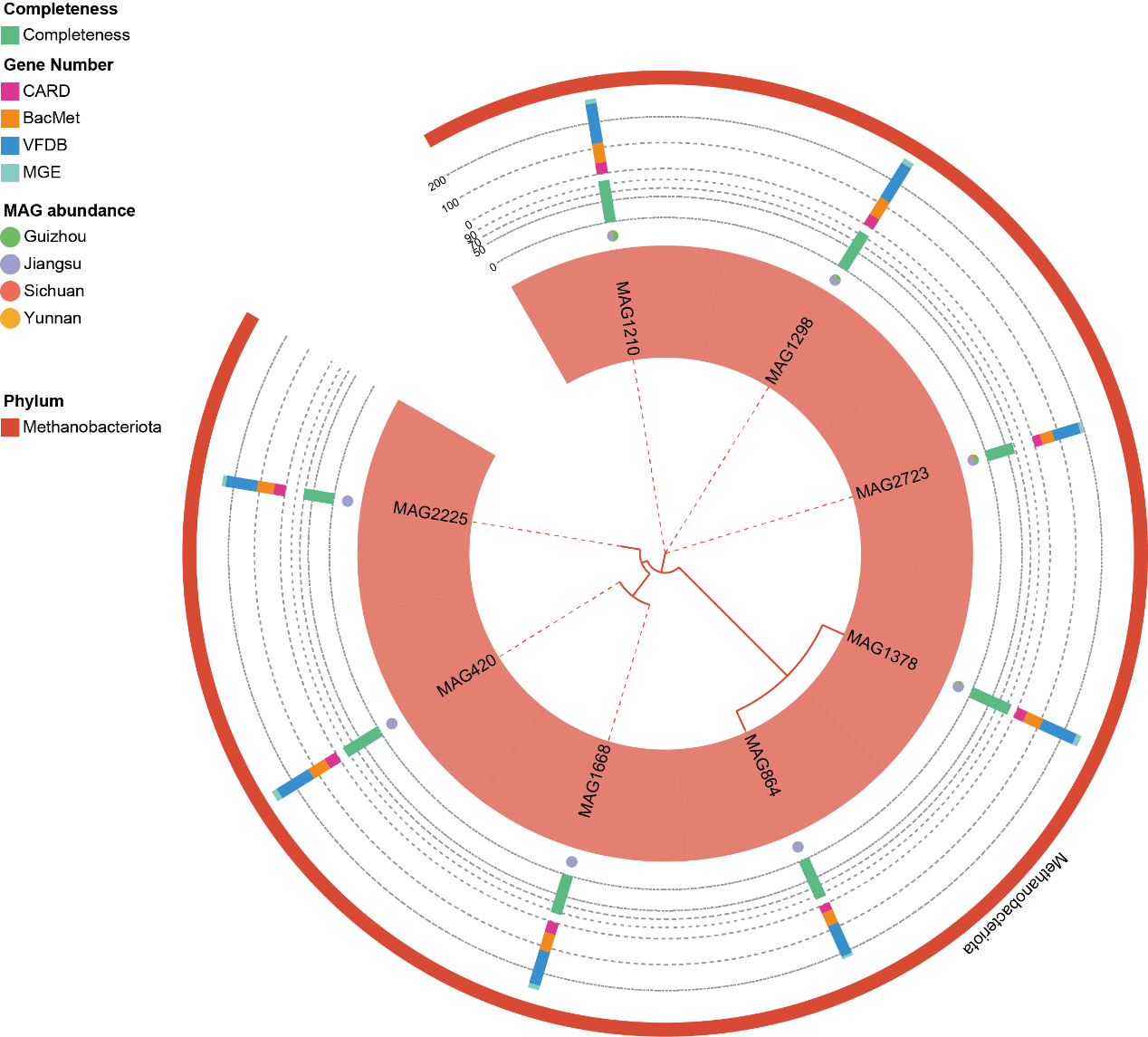
**

**Supplementary Fig. S1 Phylogenetic tree of the high-quality MAG genomes of archaeal.** The inner circle of the tree displays the names of the MAGs. Green bars indicate the completeness of the MAGs. The stacked bar chart represents the number of ARGs, MRG/BRG, VFGs, and MGEs genes corresponding to different MAG annotations in the extended CARD/BacMet/VFDB/MGE. Pie charts show the abundance proportions of different MAGs in different provinces. The outermost color bands and the background colors of the inner circle of the MAG phylogenetic tree correspond to the different phyla to which the MAGs belong.

**
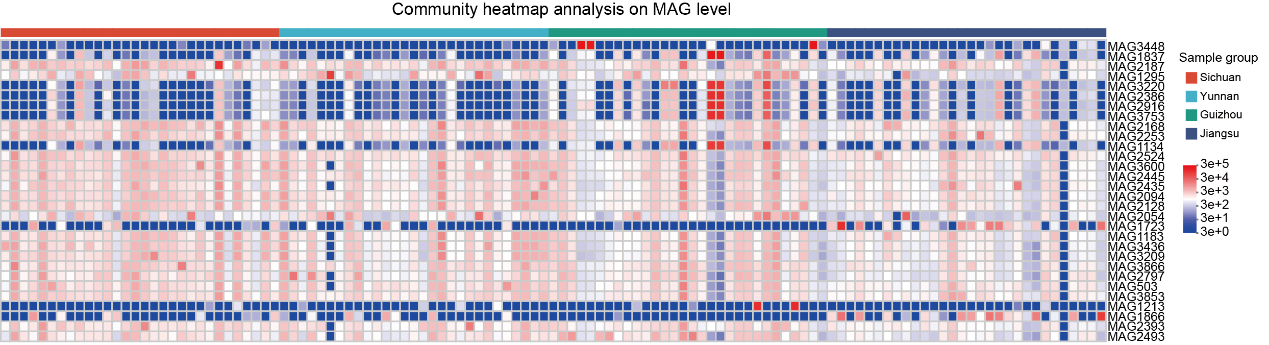
**

**Supplementary Fig. S2 The top 30 different MAGs contents in Sichuan, Yunnan, Guizhou and Jiangsu.** The transition from blue to red represents the increasing richness of MAGs.

**
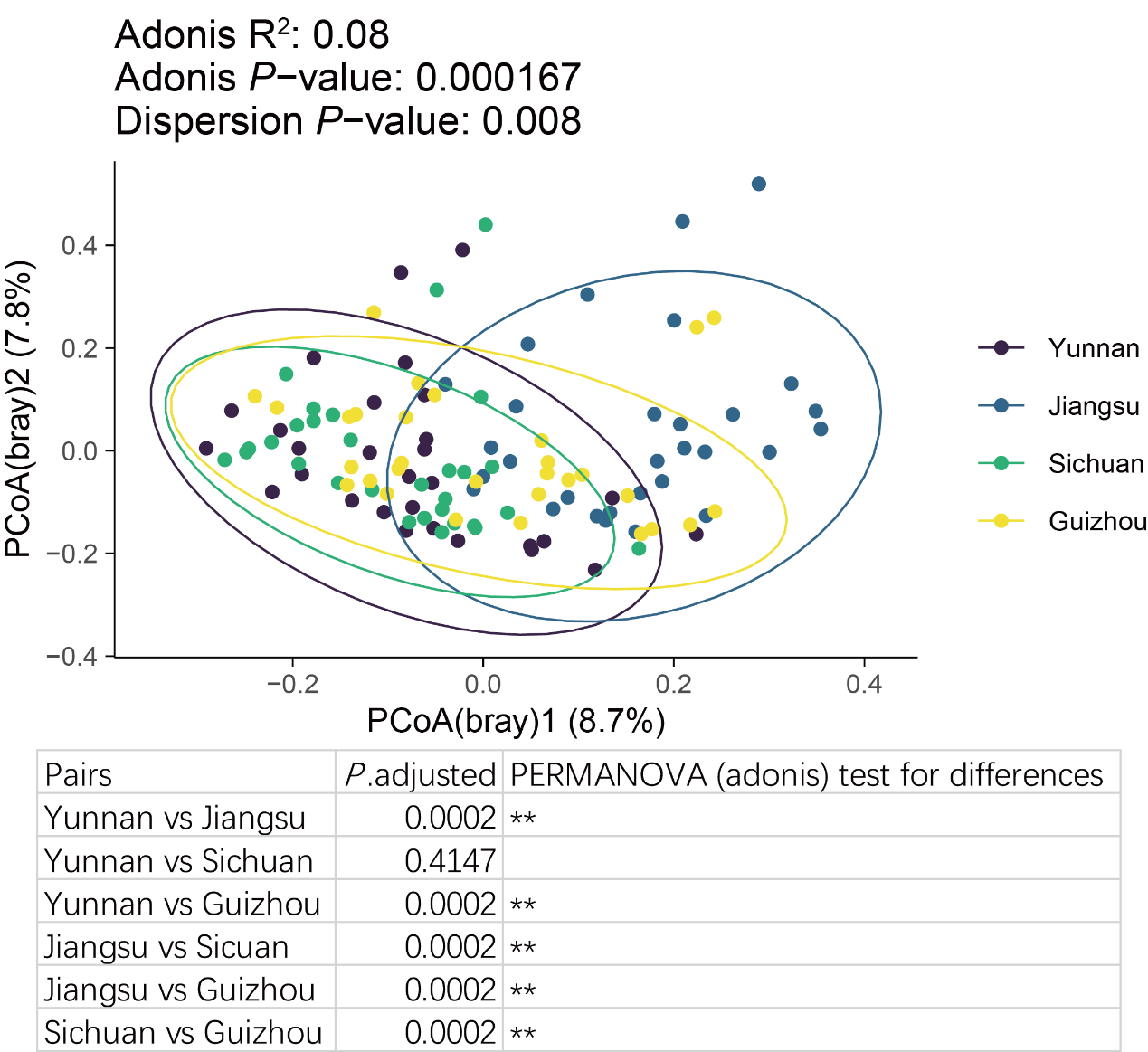
**

**Supplementary Fig. S3** **PCOA analysis of MAGs abundance in Sichuan, Yunnan, Guizhou and Jiangsu provinces.**

**
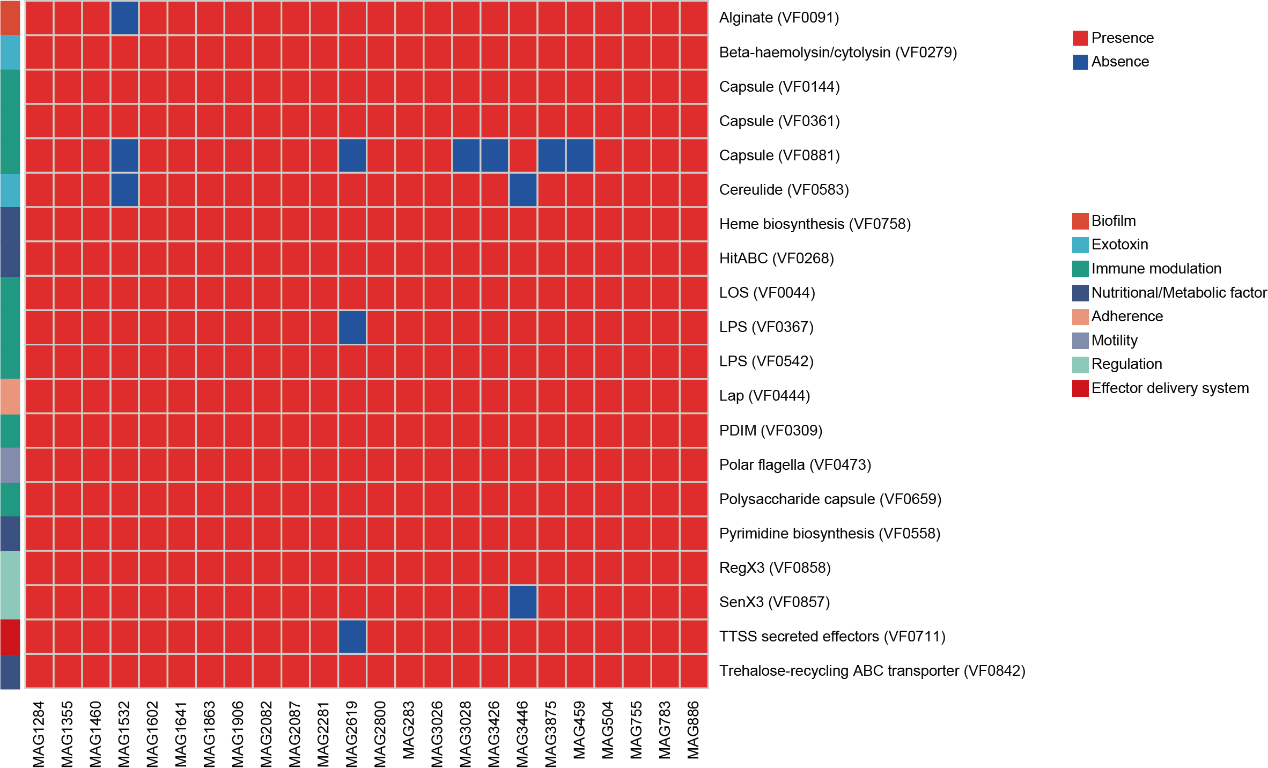
**

**Supplementary Fig. S4 Virulence gene prediction classification plot of 24 high-quality MAGs (each with > 97% completeness and 0% contamination).** The horizontal axis is the MAGID number of the genome, and the vertical axis is the virulence factor. The red legend Presence shows annotations to virulence factors, and the blue legend Absence indicates annotations not to virulence factors. Different color blocks of the vertical axis cluster tree represent different VFG categories of virulence factors.

**
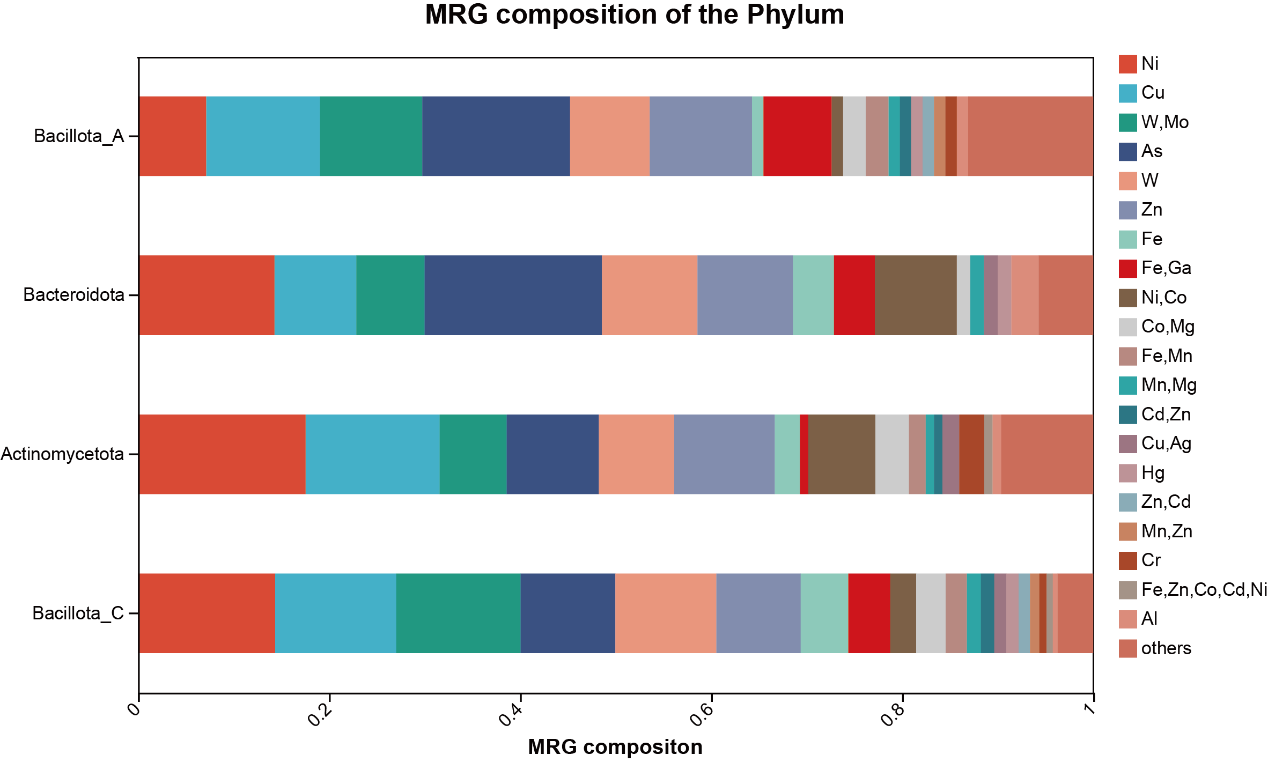
**

**Supplementary Fig. S5 The classification statistics of BacMet resistance gene predictions for 24 high-quality MAGs (each with > 97% completeness and 0% contamination).** The vertical axis represents different Phylum, and the horizontal axis represents the percentage distribution of Metal Resistance Genes annotations to the number of genes. Different color blocks in the column correspond to different Compound classifications. The legend is the Compound classification.

**
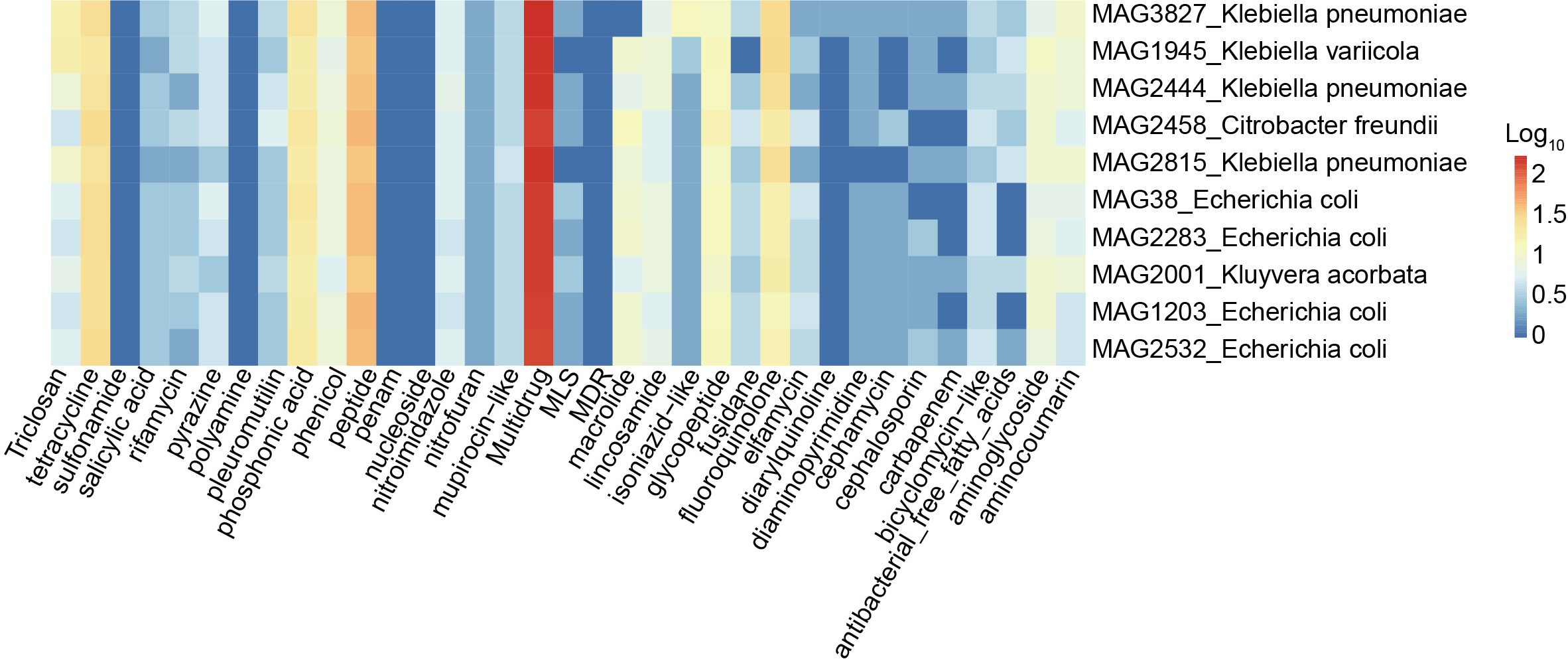
**

**Supplementary Fig. S6** **Heatmap of resistance-associated antibiotic classes in the top 10 MAG with the number of ARGs.** Colors from blue to red represent an increasing number of ARG. On the right side of the heatmap is the ID and taxonomic information of MAG, the bottom of the heatmap shows the antibiotic class.

**
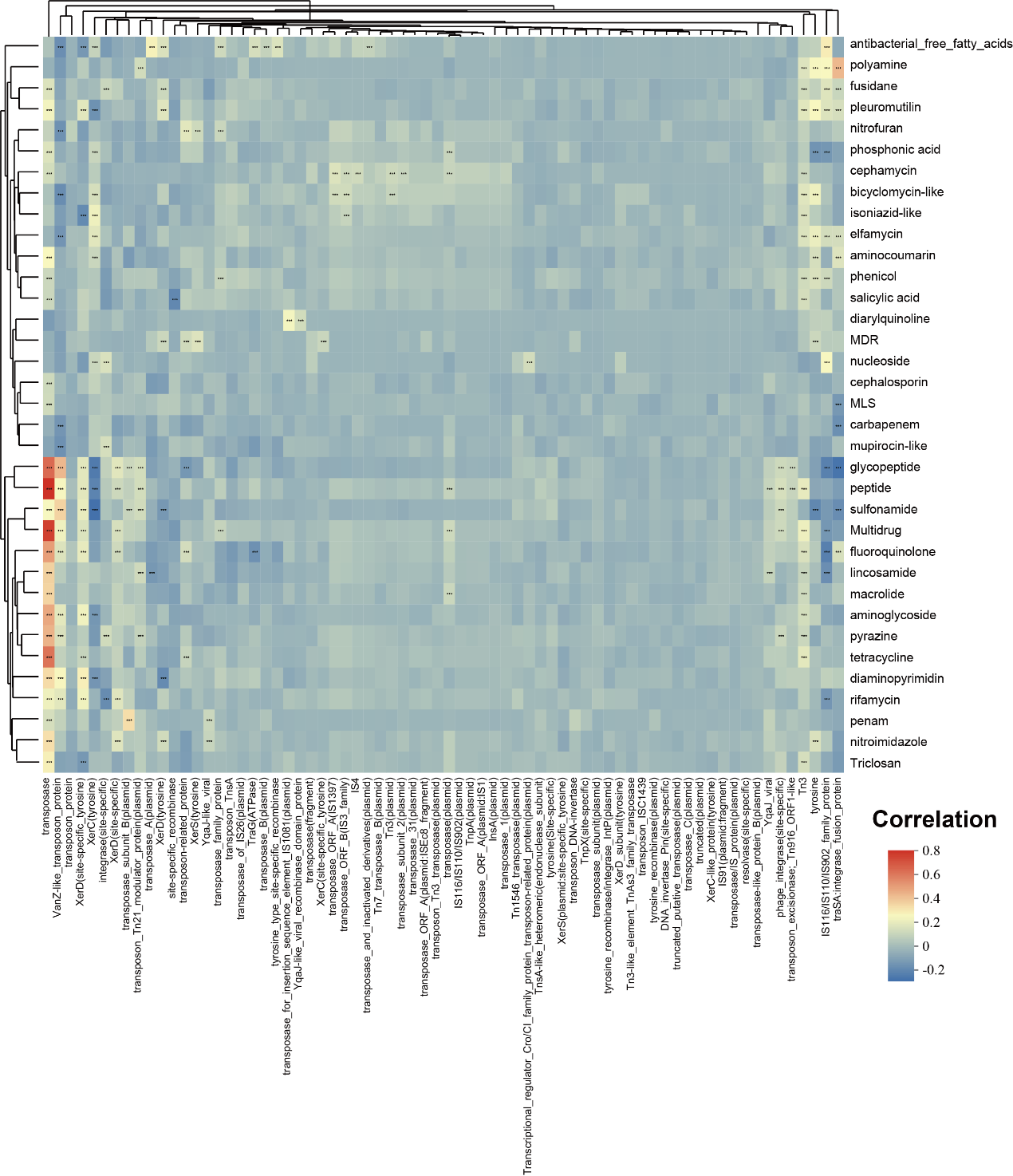
**

**Supplementary Fig. S7 The correlation heat map of Antibiotic class and MGEs.** The vertical axis is Antibiotic class and the horizontal axis is MGEs.

**
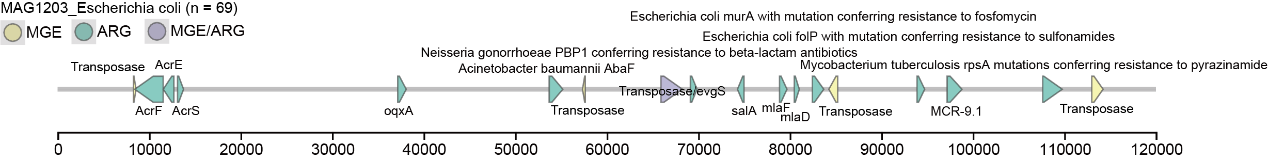
**

**Supplementary Fig. S8 The distribution of ARGs and MGEs in Escherichia coli MAG1203.** The X-axis represents the position of a gene in a continuous sequence. The direction of the arrow indicates the strand where the gene is located. The right arrow indicates the gene on the front strand, and the left arrow indicates the gene on the back strand. n=69 is the number of MAG annotations for this gene.
